# Spatiotemporal filtering modeling of hand, foot, and mouth disease: a case study from East China, 2009–2015

**DOI:** 10.1017/S0950268824001080

**Published:** 2025-04-16

**Authors:** Xi Chen, Jianbo Ba, Yuanhua Liu, Jiaqi Huang, Ke Li, Yun Yin, Jin Shi, Jiayao Xu, Rui Yuan, Michael P. Ward, Wei Tu, Lili Yu, Quanyi Wang, Xiaoli Wang, Zhaorui Chang, Zhijie Zhang

**Affiliations:** 1 Department of Epidemiology and Health Statistics, School of Public Health, Fudan University, Shanghai, China; Key Laboratory of Public Health Safety, Ministry of Education, Shanghai, China; 2 Naval Medical Center, Naval Medical University, No.880 Xiangyin Road, Yangpu District, Shanghai, China; 3 Sydney School of Veterinary Science, The University of Sydney, Camden, NSW, Australia; 4 Department of Geology and Geography, Georgia Southern University, Statesboro, GA 30460, USA; 5 Peace Center for Biostatistics, Jiann-Ping Hsu College of Public Health, Georgia Southern University, Statesboro, GA 30460, USA; 6 Beijing Center for Disease Prevention and Control; 7 Division of Infectious Disease, Key Laboratory of Surveillance and Early-Warning on Infectious Disease, Chinese Center for Disease Control and Prevention, 155 Changbai Rd, Changping District, Beijing 102206, China

**Keywords:** hand, foot, and mouth disease, spatiotemporal weight matrix, spatiotemporal filtering model, spatial epidemiology

## Abstract

Hand, foot, and mouth disease (HFMD) shows spatiotemporal heterogeneity in China. A spatiotemporal filtering model was constructed and applied to HFMD data to explore the underlying spatiotemporal structure of the disease and determine the impact of different spatiotemporal weight matrices on the results. HFMD cases and covariate data in East China were collected between 2009 and 2015. The different spatiotemporal weight matrices formed by Rook, K-nearest neighbour (KNN; *K* = 1), distance, and second-order spatial weight matrices (*SO-SWM*) with first-order temporal weight matrices in contemporaneous and lagged forms were decomposed, and spatiotemporal filtering model was constructed by selecting eigenvectors according to *MC* and the *AIC.* We used *MI*, standard deviation of the regression coefficients, and five indices (*AIC*, *BIC*, *DIC*, *R* ^2^, and *MSE*) to compare the spatiotemporal filtering model with a Bayesian spatiotemporal model. The eigenvectors effectively removed spatial correlation in the model residuals (Moran’s *I* < 0.2, *p* > 0.05). The Bayesian spatiotemporal model’s Rook weight matrix outperformed others. The spatiotemporal filtering model with *SO-SWM* was superior, as shown by lower *AIC* (92,029.60), *BIC* (92,681.20), and *MSE* (418,022.7) values, and higher *R* ^2^ (0.56) value. All spatiotemporal contemporaneous structures outperformed the lagged structures. Additionally, eigenvector maps from the Rook and *SO-SWM* closely resembled incidence patterns of HFMD.

## Introduction

Hand, foot, and mouth disease (HFMD) is an infectious disease caused by more than 20 types of enteroviruses [1, 2], among which coxsackievirus A16 (CV-A16) and enterovirus 71 (EV-71) are the most common. This disease primarily occurs in children under 5 years of age [1, 3]. A total of 11,814,830 cases of HFMD were reported in China between 2008 and 2014, with the average growth rate was 125.55/100,000 [4]. Both non-pharmaceutical interventions (such as regular disinfection) and vaccination strategies are used to control the occurrence and spread of HFMD; however, the disease remains a major public health burden in China [5].

Previous studies have indicated spatiotemporal aggregation and heterogeneity (spatially local heterogeneity and spatially stratified heterogeneity) of HFMD [4, 6–8], highlighting the importance of understanding its distribution and identifying high-risk areas and risk factors, which are prerequisites for undertaking targeted prevention and control measures to reduce the impact of HFMD [4].

Various spatiotemporal models, including the spatiotemporal autoregressive model [9, 10], spatiotemporal model with variable coefficients [11, 12], and Bayesian spatiotemporal model [2, 13, 14], have been utilized to evaluate the spatiotemporal distribution of HFMD and its associations with factors like meteorological patterns, socioeconomic status, and health conditions [15–17]. For instances, Ding et al. used an econometric panel model to explore the relationship between HFMD and meteorological factors [18]. Hong et al. constructed a monthly time-lag geographically weighted regression model to investigate spatiotemporal variations due to the effect of climatic factors on HFMD occurrence in the Inner Mongolia Autonomous Region, China [11]. Zhang et al. used a Bayesian spatiotemporal hierarchy model to identify the spatiotemporal heterogeneity (Supplementary Information S1) of HFMD [2]. However, these models suffer from mathematical complexities, intensive computation requirements, and limitations in dealing with spatial autocorrelation (e.g. the choice of spatial lag form or spatial error form in traditional autoregressive models) [19]. Additionally, they may fail to reveal the underlying spatiotemporal structure (Supplementary Information S1) and require the specification of the model’s parameter distribution [20]. Moreover, these models used only a Rook [21] or Queen [22] weight matrix in spatial autocorrelation analysis [23–25], without considering other possible configurations of weight matrix [26].

Griffith [27] proposed an eigenvector spatiotemporal filtering method to overcome the flaws in the existing models. This method first breaks down geographical and temporal relationships into a set of eigenvectors [28, 29], a subset of the eigenvectors was then incorporated into a conventional nonspatial model as covariates. The biggest advantage of this new method is that the linear combination of the selected eigenvectors can filter the spatial autocorrelation out of the observations so that the observations in the model can be treated as if they are independent [20]. The spatiotemporal filtering method can be applied to standard statistical models to construct spatiotemporal filtering models, making the modelling process simpler than that of other spatiotemporal models. In addition, the spatiotemporal filtering model allows visualization of the spatial autocorrelation of geo-referenced datasets which describes how map patterns evolve in space and time. Most importantly, compared to other models, the spatial and temporal weights can be flexibly set in a spatiotemporal filtering model to determine the optimal weight matrix better [30, 31].

In this study, we developed spatiotemporal filtering models based on HFMD case data and compared their performance against Bayesian spatiotemporal models. We explored the influence of different weight matrices on model results and visualized the eigenvector’s maps to identify potential high-risk areas for HFMD.

## Methods

### Data source

Data collection was conducted in eastern China between 2009 and 2015, focusing on HFMD cases and relevant covariates at 78 prefectural levels. HFMD case data were obtained from the national infectious disease reporting system (NIDRS) of the China CDC. To assess potential risk factors, meteorological data were retrieved from China’s National Meteorological Information Center (https://data.cma.cn). The meteorological variables included daily average temperature (°C), average humidity (%), sunlight duration (h), and average wind speed (m/s) for prefecture-level cities. Additionally, socioeconomic variables, such as GDP per capita and the number of hospitals, were collected from local official demographic yearbooks. For a comprehensive understanding of the data collection process, a more detailed description can be found in our previous study [13].

To ensure data quality and avoid multicollinearity issues, all data were summarized on a monthly basis. Covariates that displayed a correlation greater than 0.6 were scrutinized, and the one with the weaker association with HFMD case occurrence was removed from the dataset [32]. Detailed data processing is provided in the Appendix (Supplementary Information S2).

### Statistical analysis

We compared three spatiotemporal models of HFMD: the generalized linear mixed-effects model (GLMM), the spatiotemporal filtering model, and the Bayesian spatiotemporal model. We first examined the spatial correlation in the model residuals using Moran’s *I* (*MI*) to determine whether the eigenvectors could effectively remove the spatial correlation from the model residuals and then compared the model fit and accuracy between the spatiotemporal filtering model and the Bayesian spatiotemporal model.

#### Generalized linear mixed-effects model (GLMM)

The number of HFMD cases at time point *t* in each prefecture-level city *i* is denoted as 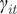 (*i* = 1, 2, …, 78). The GLMM is formulated with a logarithmic link function, and the expression is as follows:
(1)

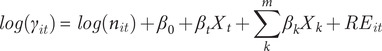

where 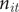 represents the population of the *i*-*th* region; 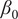 denotes the intercept term; 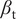 denotes the regression coefficient of the time trend; 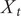 denotes the time trend (e.g. 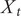 = 1, 2, …, 84); 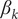 is the regression coefficient of the covariate; 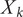 is the *k-th* covariate; and 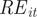 is random effect term. The variance and mean of the HFMD incidence data were unequal, which indicates that the incidence data were excessively dispersed. Therefore, a negative binomial distribution was chosen as the final link function for our GLMM based on the suggestion of the previous studies [33, 34].

#### Spatiotemporal filtering model

The spatiotemporal filtering model is based on a GLMM. The 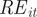 terms are divided into two components: spatial structural effect (*SSRE*) and spatial unstructured effect (*SURE*). The linear combinations of filtered eigenvectors form the SSRE terms (the screening process can be found below), while the SURE represents the model residuals.
(2)

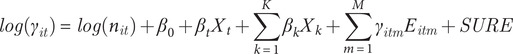

where 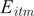 is the *m-th* eigenvector and 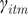 is the regression coefficient of the eigenvector.

The decomposition of a transformed connectivity matrix constitutes the core element of the spatiotemporal filtering method. More formally, decomposition yields
(3)

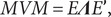

where 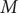 is a symmetric and idempotent projection matrix. If only the intercept is included in the construction of the projection matrix, the equation simplifies to 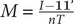 , where 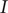 is the identity matrix and 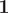 is an n × 1 one-dimensional vector. 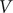 is the exogenously specified connectivity matrix (Supplementary Information S3) represented by the spatiotemporal weight matrix 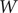 , which will be introduced below. The columns of matrix 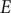 are *n* mutually independent eigenvectors obtained from the 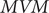 , and each eigenvector in 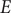 represents a distinct map pattern permitted by the units’ spatial arrangement and is associated with a certain level of spatial autocorrelation. The eigenvectors in 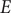 are independent and orthogonal (the advantage of orthogonality is the absence of multicollinearity) [35], which is screened first for significance before it is included in the model. 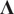 is the corresponding eigenvalue on the main diagonal of the matrix, and 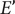 is the transposition matrix of 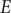 .

##### Construction of the spatiotemporal weight matrix W

Spatial weight matrices, the temporal weight matrix, and the spatiotemporal weight matrix for the spatiotemporal filtering model were constructed as follows.

First, we built four types of spatial weight matrices [34]: the Rook weight matrix, KNN (*K* = 1) weight matrix, the distance weight matrix (the list of distance vectors corresponding to the neighbouring objects was first calculated, and 1.5 times the maximum distance of neighbouring objects was specified as the distance threshold), and the second-order weight matrix. All the matrices were row normalized before modelling.

Next, we constructed a first-order temporal weight matrix to quantify the relationship between disease incidences over time. The weight between the two adjacent time periods was set to be 1 if there was a temporal dependence between the values of the two adjacent periods or if the value of a period depended exclusively on the value of the preceding period; otherwise, the weight was set to be 0. Finally, a spatiotemporal weight matrix was constructed and two sets of temporal dependence structures were specified [36].

**Figure 1.**
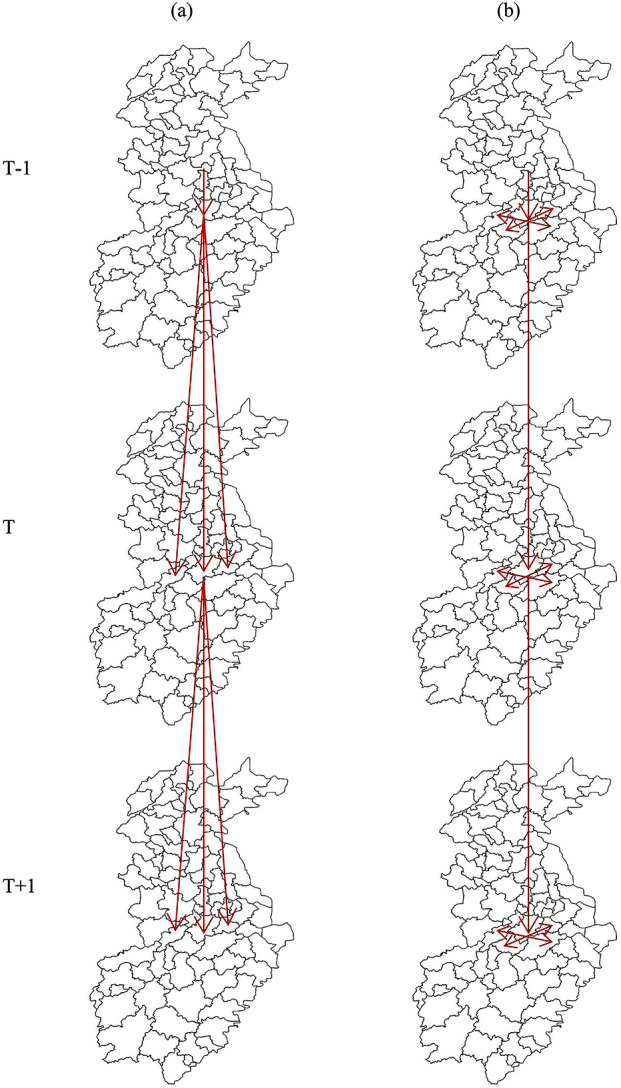
Spatiotemporal lag structure (a) and spatiotemporal contemporaneous structure (b).

1. Spatiotemporal lag structure:

The value of a position at a point in time is determined by its value at the previous point in time and the value of adjacent positions at the previous point in time (Figure 1a). The spatiotemporal time-lag dependence structure is expressed as a matrix by
(4)

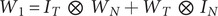

where 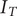 is the T*T (84*84) order unit matrix; 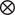 is the Kronecker; 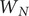 is the N*N (78*78) order spatial weight matrix; 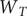 is the T*T (84*84) order temporal weight matrix; and 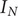 is the N*N (78*78) order unit matrix.

1. Spatiotemporal contemporaneous structure:

The value of a position at a given time point is determined by the value of all its adjacent positions and the value of that position at the previous time point (Figure 1b). In this instance, the contemporaneous spatiotemporal dependence structure is expressed as a matrix by
(5)

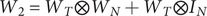



Thus, our formula (3) 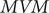 becomes either.

Lag:
(6)

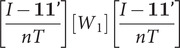

 or

Contemporaneous:
(7)

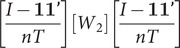



##### Screening of the eigenvectors

For the spatiotemporal filtering method, the identification and selection of the relevant eigenvectors ( 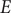 ) are decisive step before building the model [37]. The candidate set *i*∈{1,2,…,*n*} is determined by calculating *MC_i_/MC_max_* for all eigenvectors [38], where *MC* is Moran coefficient ( 
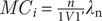
 ).


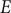
 with eigenvector, *MC_i_/MC*_max_ ≥ 0.25, enters the candidate set preliminarily. After that, a useful criterion for selecting eigenvectors from the candidate set is to combine stepwise regression and use a significance level criterion of 0.10 (the Akaike’s information criterion [*AIC*] = 0.1) [39, 40].

#### Bayesian spatiotemporal model



(8)

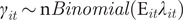

where 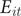 is the expected value for area i and time t. 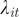 is the target estimated variable and is explained as the standard morbidity ratio (SMR) [39].
(9)

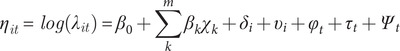



(10)

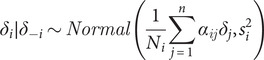

where 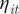 is the structured additive linear predictor; 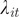 is estimated SMR of HFMD in space *i* and time *t*; 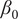 is the intercept term of the model; 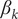 is the regression coefficient of the covariates; 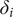 is the structured spatial influence term; 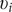 is the unstructured spatial influence term (i.e. the 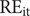 ); 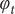 is the temporally structured effect; 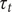 is a temporally non-structured effect; 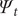 is the spatiotemporal interaction structure; and 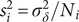 is the variance of region *i* influenced by the spatial effects of the 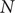 neighbouring regions, which depends on the number of its neighbouring regions 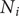 . 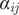 is 1 if region *i* and region *j* are adjacent, else it is 0. Here, we set four different weight matrices (Rook, KNN, distance, and second-order spatial weight matrices) to explore their effects on the model results.

The uninformative prior distribution *N* (0, 10^6^) are used for 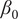 , 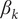 , 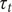 , and 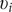 .The prior distribution of the spatial structural effect 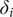 is modelled by conditional autoregression. Prior information for time effects ( 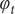 ) was modelled through a first-order autoregressive process.

#### Model evaluation

First, the spatial correlation in the residuals of the three models was examined using *MI*, where *MI* is used to determine whether spatial entities within a certain range are correlated with each other, with values distributed in [−1, 1]. Here, [0, 1] indicates a positive correlation between geographic entities, [−1,0] indicates a negative correlation, and 0 indicates no correlation [41]. Subsequently, *AIC*, Bayesian information criterion (*BIC*), deviation information criterion (*DIC*), mean square error (*MSE*), and goodness of fit (*R^2^*) were used to compare the differences between the models with different weight settings. Larger *R^2^* values and smaller *AIC*, *BIC*, *DIC*, and *MSE* values indicate better model performance[42].

R software (version 4.1.3; R Foundation for Statistical Computing, Vienna, Austria) was used for the analysis, and the INLA, dplyr, spdep, and lme4 packages were utilized for data processing, matrix creation, and model development. QGIS (version 3.1.4; QGIS Development Team) was used to create maps.

## Results

### Spatial and temporal characteristics of HFMD in eastern China

**Figure 2.**
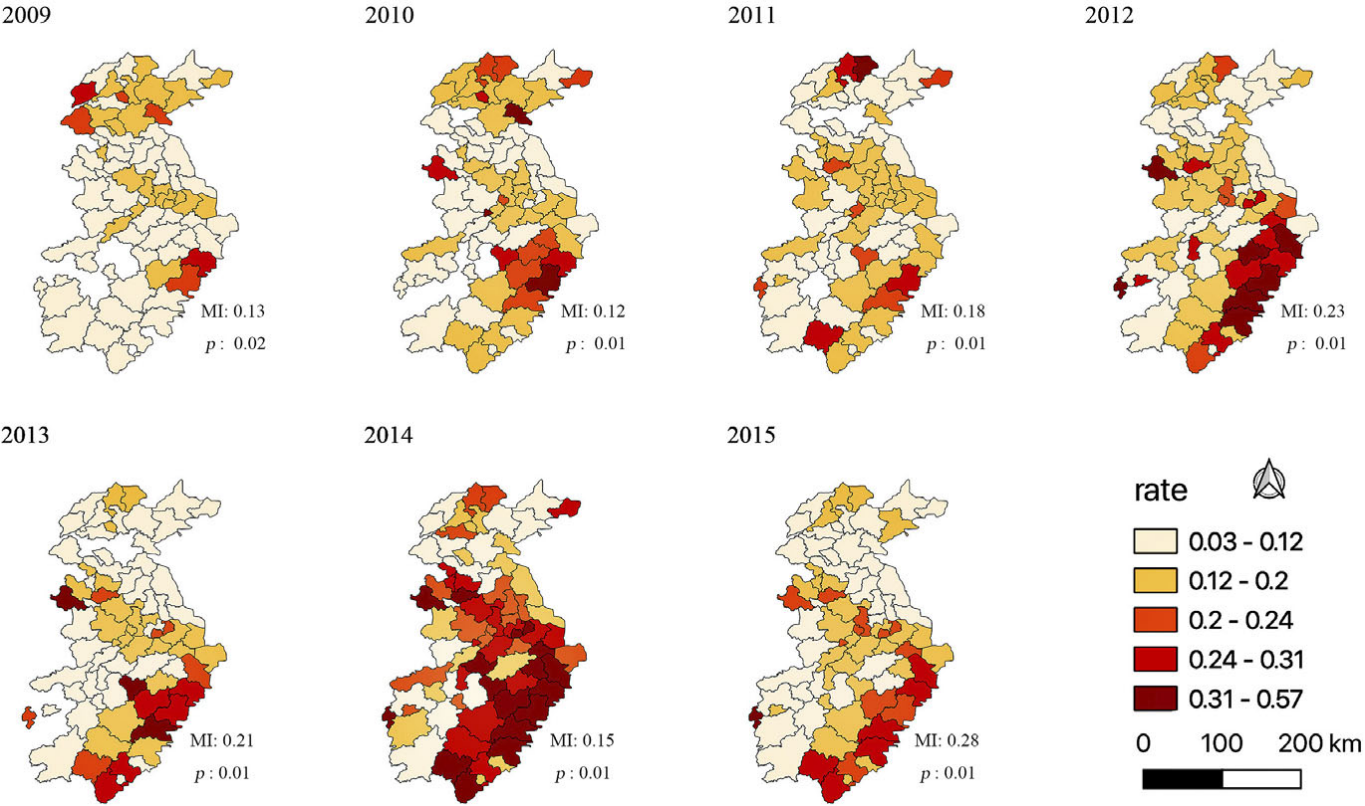
Incidence maps of HFMD in east Mainland China, 2009–2015.

Between 2009 and 2015, a total of 4,058,702 HFMD cases were reported. The incidence rates varied across space, but the disease was concentrated mainly in the south-eastern region of the study area. The incidence rate was also increasing over time but the rate was lower in 2015 than 2014 (Figure 2). The time-series plot of the HFMD incidence rate further revealed distinct seasonal patterns, indicating major incidence peaks occurring from May to July and minor peaks from September to November each year (Supplementary Information S4). Meanwhile, the incidence of HFMD in the East China showed spatially local heterogeneity (Supplementary Information S5). These findings suggest that HFMD is both temporally and spatially correlated and heterogeneous, necessitating further spatiotemporal analysis.

### Model specification, assessment, and comparison

**Figure 3.**
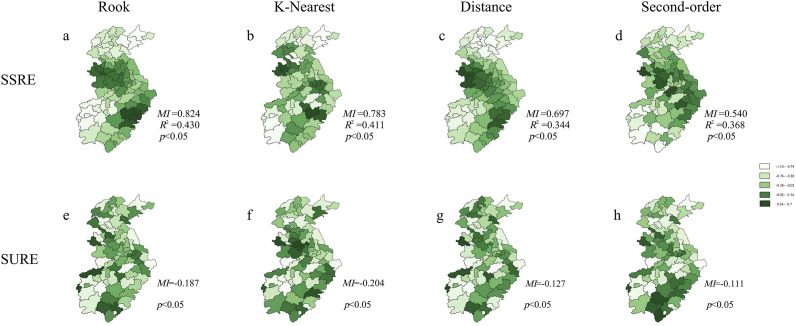
SSRE and SURE terms after decomposition of random effect terms. *The values of the colour in the graph are relative sizes.

The *RE* term of the GLMM was decomposed into SSRE and SURE, as illustrated in Figure 3. The SSRE showed spatial aggregation, whereas the SURE had no obvious spatial distribution pattern.

The *MI* of the original GLMM’s residuals demonstrated spatial correlation and was statistically significant (*p* < 0.05). The residuals of the spatiotemporal filtering model were effectively free of spatial correlation after adding the eigenvectors (*p* > 0.05) (Table 1).

**Table 1.** Comparison of the results from the spatiotemporal filtering models and Bayesian spatiotemporal models

	Spatiotemporal filtering model								Bayesian spatiotemporal model			
	R^g^-C^k^	R-L^l^	K^h^-C	K-L	D^i^-C	D-L	S^j^-C	S-L	R	K	D	S
*MI-NON^a^*	0.29	0.29	0.25	0.25	0.22	0.22	0.07	0.07	0.22	0.18	0.23	0.07
*P-NON* ^b^	0.01	0.01	0.03	0.03	0.01	0.01	0.04	0.04	0.01	0.09	0.01	0.05
*N(initial)* ^c^	4,158	3,188	1906	1,691	2,439	1733	2,425	1918	/	/	/	/
*N(added)* ^d^	249	213	233	197	81	122	86	63	/	/	/	/
*MI* ^e^	0.09	0.14	0.17	0.15	0.01	0.03	0.09	0.05	/	/	/	/
*P* ^f^	0.24	0.14	0.11	0.12	0.45	0.37	0.22	0.33	/	/	/	/
*AIC*	92,196.90	92,261.90	92,336.60	92,345.90	92,064.00	92,209.80	92,029.60	92,219.70	92,485.20	92,425.90	92,508.44	92,509.90
*BIC*	93,954.90	93,775.50	93,986.00	93,751.00	92,681.70	92,949.60	92,681.20	92,715.10	92,678.59	92,618.91	92,701.69	92,703.13
*R* ^2^	0.56	0.56	0.57	0.56	0.56	0.55	0.56	0.55	0.24	0.24	0.24	0.24
*DIC*	/	/	/	/	/	/	/	/	92,020.78	92,020.82	92,020.65	92,020.53
*MSE*	419,249.40	420,002.30	422,758.80	419,759.90	422,841.70	420,588.10	418,022.70	418,185.30	6,505,859.00	6,506,572.00	6,506,679.00	6,506,667.00

a *MI-NON: MI* value of generalized linear mixed-effects model (GLMM) residuals.

b *P-NON*: *P* value of *MI* value in GLMM residuals.

c *N (initial):* the number of eigenvectors with *MIi/MImax* ≥ 0.25.

d *N (added):* the number of eigenvectors after stepwise regression screening.

e *MI: MI* value of spatiotemporal filtering model residuals.

f *P*: *P* value of *MI* value in spatiotemporal filtering model residuals.

g R: Rook.

h K: K-nearest neighbour (KNN; *K* = 1).

i D: Distance.

j S: Second-order.

k C: Contemporaneous.

l L: Lagging.

The Bayesian spatiotemporal model with Rook weight matrix performed better based on the *DIC* and *MSE* values. The spatiotemporal filtering model with second-order contemporaneous weight matrix outperformed other models, as evidenced by lower *AIC*, *BIC*, and *MSE* values, and higher *R* ^2^ value. Furthermore, all spatiotemporal contemporaneous structures were superior to the models with lagged structures (Table 1).

Comparing the results of the optimal spatiotemporal filtering model with those of the Bayesian spatiotemporal model, the *AIC* and *MSE* values of the former model were 92,029.6 and 418,022.7, respectively, which were lower than those of the latter model. In addition, the *R* ^2^ value of the former model was about 30% higher than that of the latter model. These results suggest that the spatiotemporal filtering model performed better than the Bayesian spatiotemporal model (Table 1).

### Model coefficients

Table 2 shows that the model coefficients from the spatiotemporal filtering models and Bayesian spatiotemporal models were overall consistent, sunlight and average humidity were negatively related to HFMD, whereas average wind speed and average temperature were positively associated with HFMD. However, an important observation is that the standard deviations of all the coefficients in the spatiotemporal filtering models were smaller compared to those in the Bayesian spatiotemporal models. This indicates a higher precision of estimation in the spatiotemporal filtering models. The smaller standard deviations suggest that the coefficients estimated by the spatiotemporal filtering models are more reliable and less affected by variability in the data.

**Table 2.** Comparison of coefficients of the model

	Spatiotemporal filtering model								Bayesian spatiotemporal model			
	R-C	R-L	K-C	K-L	D-C	D-L	S-C	S-L	R	K	D	S
*β*	−10.05 (0.22) ^a^	−10.15 (0.22) ^a^	−10.15 (0.22) ^a^	−10.12 (0.22) ^a^	−10.23 (0.22) ^a^	−10.09 (0.22) ^a^	−10.24 (0.22) ^a^	−10.12 (0.22) ^a^	2.445 (0.23)	2.484 (0.24)	2.475 (0.24)	2.473 (0.24)
Average humidity	−0.09 (0.02) ^a^	−0.08 (0.02) ^a^	−0.07 (0.02) ^a^	−0.08 (0.02) ^a^	−0.06 (0.02) ^a^	−0.07 (0.02) ^a^	−0.05 (0.02) ^a^	−0.07 (0.02) ^a^	−0.07 (0.02)	−0.07 (0.02)	−0.07 (0.02)	−0.07 (0.02)
Average wind speed	0.08 (0.03) ^a^	0.09 (0.03) ^a^	0.09 (0.03) ^a^	0.10 (0.03) ^a^	0.10 (0.03) ^a^	0.09 (0.03) ^a^	0.09 (0.03) ^a^	0.09 (0.03) ^a^	0.09 (0.03)	0.09 (0.03)	0.09 (0.03)	0.09 (0.03)
Hours of sun	−0.04 (0.01) ^a^	−0.04 (0.01) ^a^	−0.04 (0.01) ^a^	−0.05 (0.01) ^a^	−0.04 (0.01) ^a^	−0.04 (0.01) ^a^	−0.05 (0.01) ^a^	−0.04 (0.01) ^a^	−0.04 (0.01)	−0.04 (0.01)	−0.04 (0.01)	−0.04 (0.01)
Average temperature	0.07 (0.01)^a^	0.07 (0.01)^a^	0.07 (0.01)^a^	0.07 (0.01)^a^	0.07 (0.01)^a^	0.07 (0.01)^a^	0.07 (0.01)^a^	0.07 (0.02)^a^	0.07 (0.02)	0.07 (0.02)	0.07 (0.02)	0.07 (0.02)
Average GDP	0.01 (0.01)^a^	0.01 (0.01)	0.01 (0.01)	0.01 (0.01)	0.01 (0.01)	0.01 (0.01)	0.01 (0.001)	0.01 (0.02)	−0.01 (0.02)	−0.01 (0.02)	−0.01 (0.02)	−0.01 (0.02)
Hospital number	−0.02 (0.05)	−0.03 (0.05)	−0.02 (0.05)	−0.01 (0.05)	−0.01 (0.06)	−0.01 (0.06)	0.01(0.053)	0.01 (0.061)	0.45 (0.06)	0.45 (0.06)	0.44 (0.06)	0.45 (0.06)

a indicates statistical significance (*p* < 0.05).

### Exploration of map patterns based on eigenvectors

**Figure 4.**
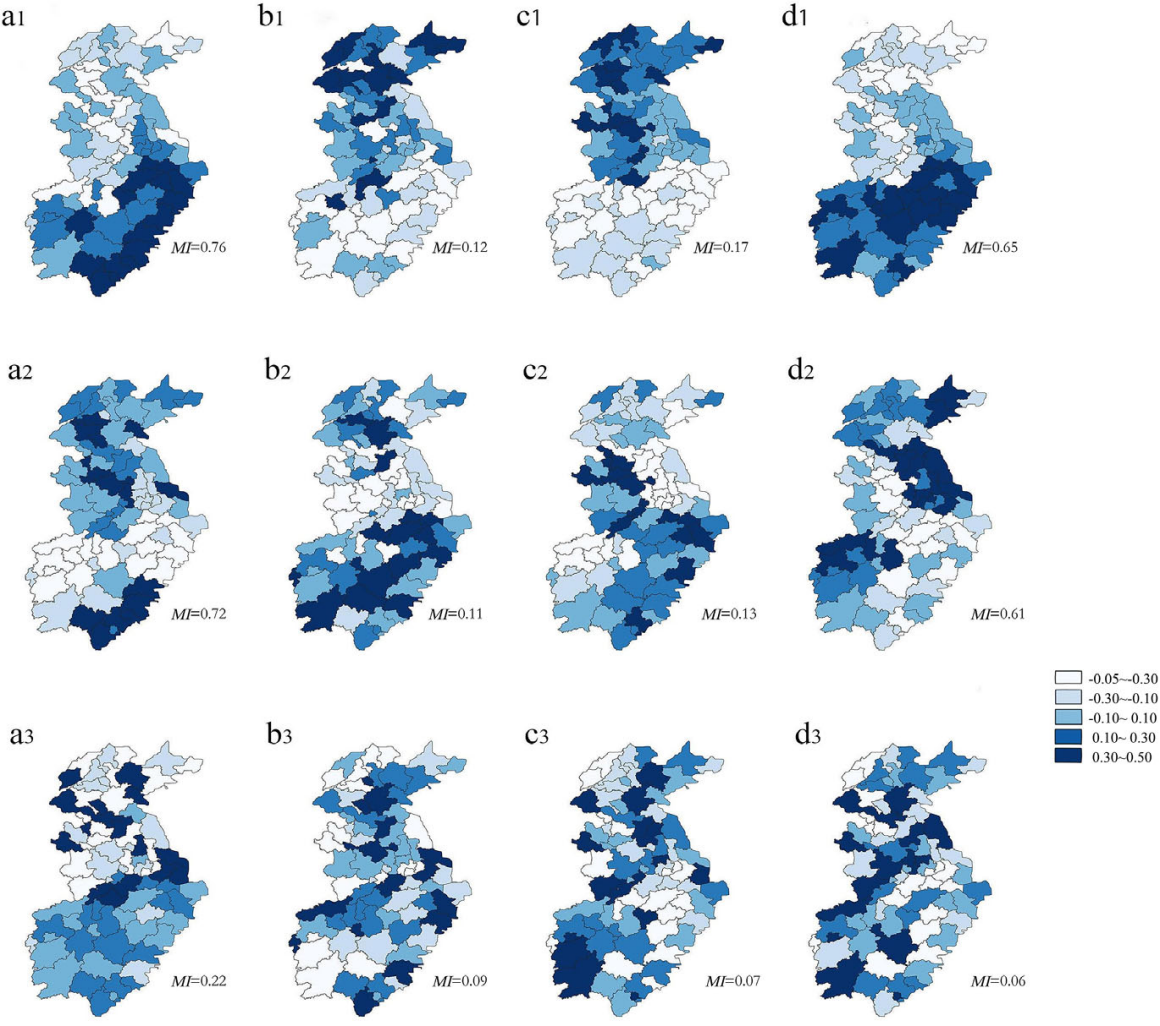
Eigenvector decomposition of the spatiotemporal simultaneous structure matrix. (a), (b), (c), and (d) represent the Rook, KNN (*K* = 1), distance, and second-order matrices, respectively, where (a1), (b1), (c1), and (d1) represent the first eigenvectors; (a2), (b2), (c2), and (d2) are the second eigenvectors; and (a3), (b3), (c3), and (d3) are the last eigenvectors. * The colours in the figure represent map modes with different *MI* values. The darker the colour, the more concentrated the place is. All values are greater than 0.

Figure 4 shows three statistically significant eigenvectors generated by models using different spatiotemporal matrices. These eigenvectors capture varying levels of spatiotemporal information. The first two eigenvectors contain the most spatiotemporal information, while the last eigenvector represents the least spatiotemporal information. The mapped geographical patterns are related to the eigenvectors at different geographical scales. While the first two eigenvector maps show a regional (larger) clustering pattern with a north–south orientation, the last eigenvector map shows a local (smaller) clustering pattern with much less visible orientation.

Moreover, on the first eigenvector maps with the Rook and the second-order matrices (Figure 4: a1, d1) and on the HFMD incidence map (Figure 2), HFMD appeared to be concentrated in the south-eastern region of the study area.

## Discussion

In this study, spatiotemporal filtering models were constructed to analyse the HFMD incidence in East China. The eigenvectors in these models effectively removed the spatial correlation from the residuals to better reveal the underlying spatiotemporal structure of the HFMD data. This information can help identify the risk factors and design effective interventions and control programs. We also compared the spatiotemporal filtering models and GLMM and the Bayesian spatiotemporal models. Based on the evaluation index, model coefficients, and the standard deviations of the model coefficients, goodness of fit, and precision of estimation, the spatiotemporal filtering models overall overperformed the Bayesian spatiotemporal models and therefore can be considered a more optimal approach to conduct the spatiotemporal analysis of HFMD in East China.

Identifying appropriate spatial weight matrices that can best represent the spatial relationship between the outcomes in the neighbouring spatial units is a critical step to investigate the spatial autocorrelation of HFMD [43]. Different from most of the previous studies that used only one spatial weight matrix, we applied four different spatial weight matrices to examine how and to what extent the spatial weight matrices could influence the model results. We also extended the investigation to include spatiotemporal weight matrices in the models. Our findings are as follows. First, the Rook spatial weight matrix was the best choice for the Bayesian spatiotemporal models, and this conclusion is consistent with some earlier findings [34, 43]. HFMD spreads mainly through close contact, so people nearby are more susceptible to the disease. Second, expanding to the spatiotemporal matrix, the second-order weight matrix performed the best in the spatiotemporal filtering models. This might be because the spatiotemporal weight matrix captures the spatiotemporal interactions, whereas in the Bayesian spatiotemporal model, only the spatial weight matrix can be used. Third, the spatiotemporal contemporaneous structure was better than the lagging structure; this might be explained by the relatively short incubation period of HFMD [44] (approximately 5–7 days). Therefore, the monthly HFMD incidence was more significantly influenced by contemporaneous structure than by lag structure. Our eigenvector plots also showed that the Rook and second-order weight matrices were more consistent with the observed incidence of HFMD, suggesting that in our study area, there may be slight differences between the Rook and the second-order weight matrices. The first two eigenvectors of each matrix in Figure 4 contain relatively more spatiotemporal information and show clustering patterns for areas in the north–south direction (larger); whereas the last eigenvector represents the least spatiotemporal information and shows clustering patterns for places where the direction is not obvious (smaller). The map pattern that contains the most spatiotemporal information is most likely the major spatial distribution (e.g. east–west or north–south structure). This also verifies our conjecture and suggests that our eigenvector visualization can reveal the potential important areas that are the priorities for disease prevention and control.

Compared to GLMM and the Bayesian spatiotemporal model, the spatiotemporal filtering model could reveal underlying spatial patterns by extracting and visualizing eigenvectors. It detected spatial structures and patterns at finer spatial scales. Such information could potentially be used to reveal directions of disease spread and inform disease control priority, although the underlying mechanism of the discerned spatiotemporal structure and pattern will need to be investigated in separate studies.

The eigenvectors decomposed from the spatiotemporal filtering models are independent of each other; they can easily be applied to traditional statistical models to avoid the mathematical intractability issue that many traditional statistical models such as the spatial autoregression model suffer from. In addition, the spatiotemporal filtering models do not rely on the Markov chain Monte Carlo simulation as do Bayesian models [19], which make them require much less computational resources. In addition, several studies have begun to explore the spatiotemporal filtering method in practical applications. For example, using the eigenvector spatial filtering method, Zhang et al. attempted to estimate ground PM2.5 concentrations [45], and Chen et al. built a spatiotemporal eigenvector filtering model based on population flow to examine the spatiotemporal pattern of health risk factors associated with COVID-19 [36] and effectively improved the model prediction accuracy. Results of the current study indicate that the spatiotemporal filtering model offers a promising alternative to traditional complex spatiotemporal models in disease-related applications.

In this study, we found that there is global spatial correlation and spatial heterogeneity in the incidence of HFMD in East China. The risk of HFMD in East China is influenced by meteorological factors (average temperature, average humidity, sunshine hours, and average wind speed) and economic factors (GDP), as indicated by our modelling results. So, we measured spatially stratified heterogeneity of the model’s residuals; we found that the heterogeneity became rather weak, indicating that the spatially stratified heterogeneity of HFMD may be attributable to the included factors in our model. Hence, the models used in this study are reasonable.

However, this study had several limitations. First, we compared model results of a few configurations of spatial weight matrices that are based on geometric adjacency; however, adjacency can also be based on the various geographic characteristics (demographic, socioeconomic, etc.), which should be explored in future studies. Also, in subsequent studies, it is necessary to investigate more innovative computational methods to further improve the efficiency of calculation (e.g. by using parallel computing). Second, how and to what extent the number of eigenvectors impact model results also require detailed studies.

## Conclusions

The spatiotemporal filtering model is an effective spatiotemporal analysis method that can be used as a suitable alternative to traditional complex spatiotemporal models for practical application in the control of HFMD. More importantly, the generated eigenvectors can be used to reveal the potential direction of disease spread, which has not been possible with previous spatiotemporal models. However, the appropriate spatial and spatiotemporal weight matrices must be carefully considered.

## Supporting information

Chen et al. supplementary material Chen et al. supplementary material

## Data Availability

The data that support the findings of this study are available on request from the corresponding author (Zhijie Zhang). The data are not publicly available due to restrictions (e.g. their containing information that could compromise the privacy of research participants).

## Supplementary material

For supplementary material accompanying this paper visit http://doi.org/10.1017/S0950268824001080. click here to view supplementary material
