# Supplementary material for: Spatiotemporal filtering modeling of hand, foot, and mouth disease: a case study from East China, 2009–2015
Source: Epidemiol Infect. 2025 Apr 16;153:e61. doi: 10.1017/S0950268824001080 (PMC12041904; doi:10.1017/S0950268824001080)
Supplement: Chen et al. supplementary material [file S0950268824001080sup001.docx]

**Appendix information:**

**S1 Definitions of “spatiotemporal heterogeneity”, “spatiotemporal structure”**

"Spatiotemporal heterogeneity" and "spatiotemporal structure" do not have very clear definitions.

Existing literature points out that the spatial heterogeneity of diseases refers to the inhomogeneity of the spatial distribution of diseases and their complexity, and some literature also indicates spatial heterogeneity by *MI*<0, while temporal heterogeneity refers to the diversity of disease trends over time. Therefore, the spatiotemporal heterogeneity means the variability in the geospatial and temporal distribution of the same disease as a reflection of the intensity of the disease and the distribution of its influences and interactions at a specific time and geographic location.

While for spatial structure, it refers to the spatial distribution characteristics of the disease, such as certain clustered distributions, map patterns of high east and low west or low east and high west. Temporal structure refers to the trend of certain features or attributes of a disease over time. Hence, the spatiotemporal structure means the distribution of certain attributes or phenomena in different spaces and the different patterns of arrangement or linkages over time.

**S2 Covariates included in the model**

**S2.1 Definition of the variables**

1. avg Humid: Average humidity (per week);
2. avg Temp: Average temperature (per week);
3. sun Hour: Hours of sun (per week);
4. n: Number of cases of hand-foot-mouth disease
5. school Elementary: Number of elementary school;
6. parkNum: Number of Parks;
7. hospital Num: Number of Hospitals(Hospital number);
8. school Kindergarten: Number of kindergartens
9. avg WindSpeed: Average wind speed (per week);
10. avg Gdp: Average GDP (per capita);

**S2.2 Fig A. Collinearity tests**


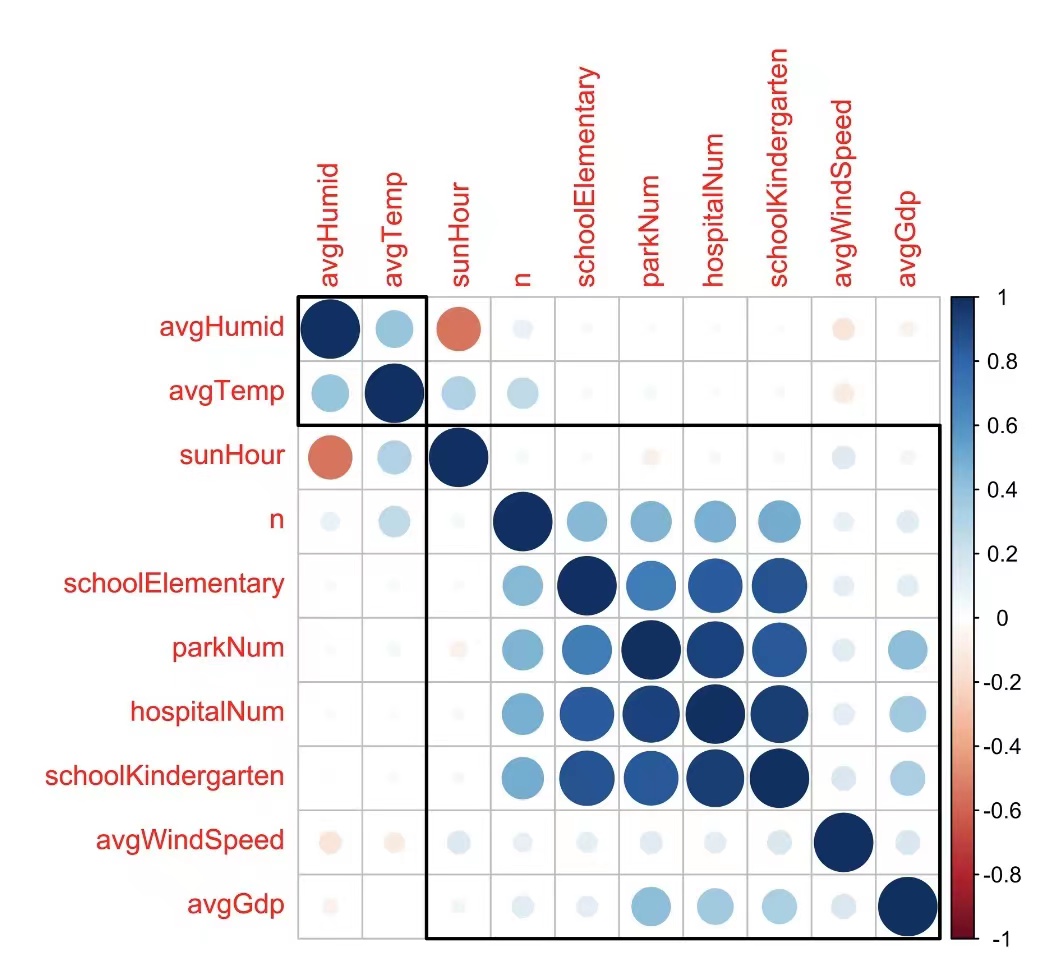


**S2.2 Table A: Impact factors collinearity diagnostics**

In this study, variables leading to Spearman correlation coefficient greater than 0.6 were not included in the final analysis model.

|  | n | avg Humid | avg Wind speed | sun Hour | avg Temp | avg  Gdp | hospital Num | park Num | school kindergarten | school Elementary |
| --- | --- | --- | --- | --- | --- | --- | --- | --- | --- | --- |
| n | 1.00 | 0.10 | 0.10 | 0.05 | 0.27 | 0.13 | 0.48 | 0.47 | 0.50 | 0.45 |
| avg Humid | 0.10 | 1.00 | -0.13 | -0.54 | 0.39 | -0.07 | -0.02 | 0.02 | -0.01 | 0.03 |
| avg Windspeed | 0.10 | -0.13 | 1.00 | 0.15 | -0.11 | 0.17 | 0.13 | 0.14 | 0.16 | 0.11 |
| sun Hour | 0.05 | -0.54 | 0.15 | 1.00 | 0.32 | 0.06 | -0.03 | -0.07 | -0.03 | -0.02 |
| avg Temp | 0.27 | 0.39 | -0.11 | 0.32 | 1.00 | -0.01 | 0.02 | 0.04 | 0.03 | 0.03 |
| avg Gdp | 0.13 | -0.07 | 0.17 | 0.06 | -0.01 | 1.00 | 0.37 | 0.43 | 0.34 | 0.12 |
| hospital Num | 0.48 | -0.02 | 0.13 | -0.03 | 0.02 | 0.37 | 1.00 | 0.92 | 0.94 | 0.84 |
| park Num | 0.47 | 0.02 | 0.14 | -0.07 | 0.04 | 0.43 | 0.92 | 1.00 | 0.84 | 0.70 |
| school Kindergarten | 0.50 | -0.01 | 0.16 | -0.03 | 0.03 | 0.34 | 0.94 | 0.84 | 1.00 | 0.86 |
| school Elementary | 0.45 | 0.03 | 0.11 | -0.02 | 0.03 | 0.12 | 0.84 | 0.70 | 0.86 | 1.00 |

**S3 Definitions of “connectivity matrix”**

connectivity matrix:

The connectivity between each pair of nodes in a network is usually represented as a two-dimensional matrix. In this matrix, each row and column correspond to a different node, and the matrix element located at the intersection of the *i_th_* row and *j_th_* column represents the information about the connectivity between node *i* and node *j*. This matrix representation is often referred to as connectivity matrix.

$$C_{ij}\left\{ \begin{aligned} C_{11} C_{12}\ldots C_{1N} \\ C_{21} C_{22}\ldots C_{2N} \\ \ldots\\ C_{N1} C_{N2}\ldots C_{NN} \end{aligned} \right.$$

**S4 Time series chart of East China**

Figure A: Time series chart of the number of morbidity


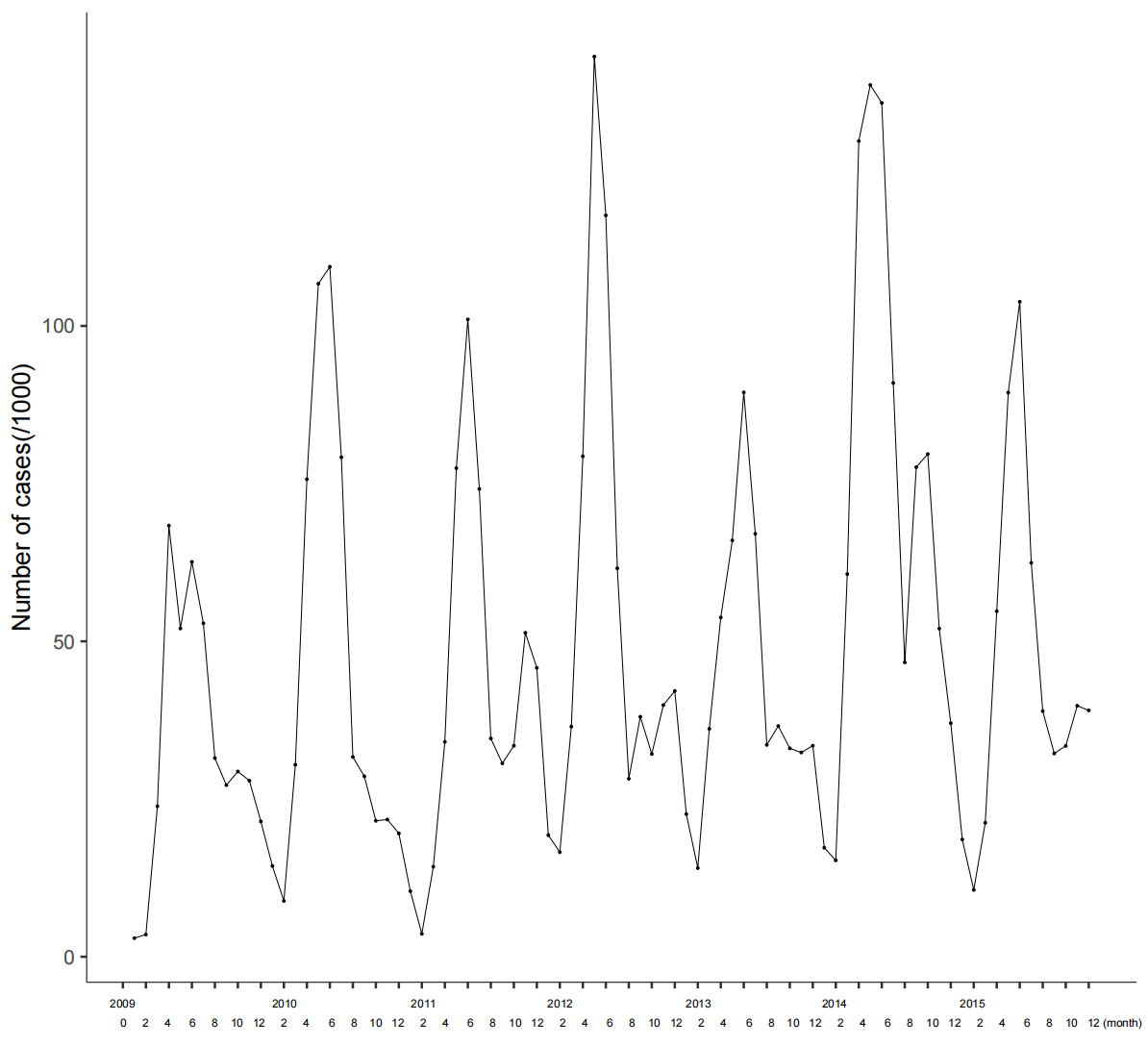


**S5 Local Indicators of Spatial Association(LISA)**


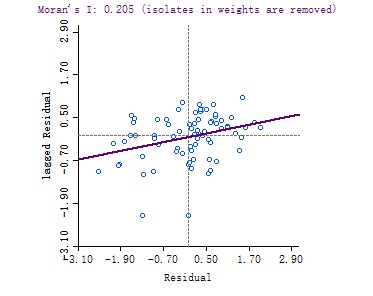


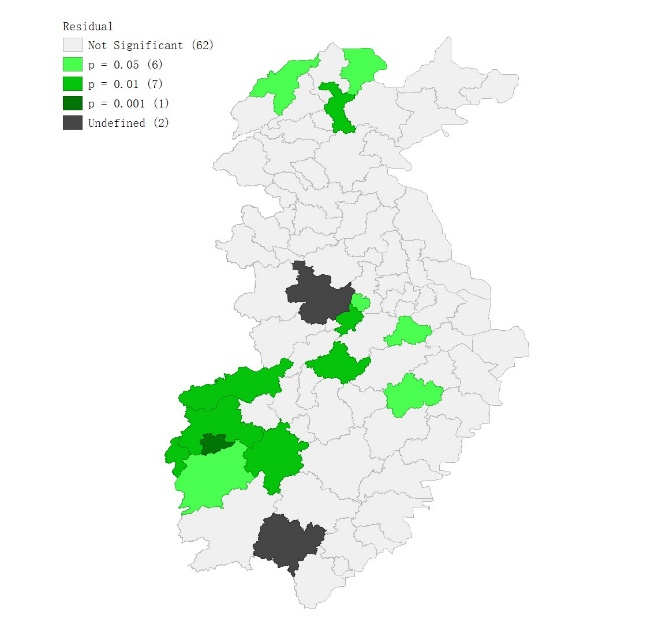

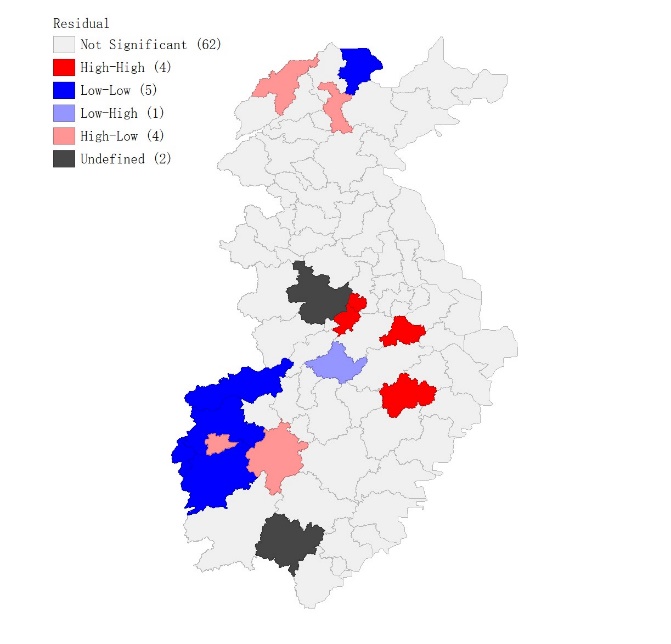


**S6 Further comparison of results between spatiotemporal filtering models and Bayesian spatiotemporal models.**

| **Model** | | **LR** | ***K*** | ***δ*** |
| --- | --- | --- | --- | --- |
| **Null** |  | -46087.6 |  |  |
| **Spatiotemporal filtering model** | R-C | -45839.4 | 259 | 52411.09 |
|  | R-L | -45877.4 | 254 | 52458.13 |
|  | K-C | -45925.3 | 243 | 52629.99 |
|  | K-L | -45966 | 207 | 52442.99 |
|  | D-C | -45941.0 | 91 | 52635.15 |
|  | D-L | -45995.9 | 109 | 52494.70 |
|  | S-C | -45918.8 | 96 | 52334.36 |
|  | S-L | -46036.8 | 83 | 52344.53 |
| **Bayesian spatiotemporal model** | R | -46160.54 | 82 | 82206461.6 |
|  | K | -46131.05 | 82 | 82206472.9 |
|  | D | -46172.22 | 82 | 82206474.6 |
|  | S | -46172.96 | 82 | 82206461.4 |

* ***LR***: the likelihood; ***K***: the number of parameters; ***δ***: error
